# Robustness of Tumor Control Against Intrafraction Patient Motion in Lung Stereotactic Body Radiation Therapy

**DOI:** 10.1016/j.adro.2026.102064

**Published:** 2026-04-17

**Authors:** Yohan A. Walter, Chiachien J. Wang, Daniel B. Speir, William E. Burrell, Mitchell Wolden, Olivia G. Moncrief, Megan M. Rodrigues, James C. Henry, Carlos D. Palomeque, Anne N. Hubbard, Philip F. Durham, Troy D. Jacobs, Bethany L. Broekhoven, Joseph P. Dugas, Hsinshun T. Wu

**Affiliations:** a Department of Radiation Oncology, Willis Knighton Cancer Center, Shreveport, Louisiana; b Department of Clinical Research, University of Jamestown, Fargo, North Dakota; c Medical School, Louisiana State University Health Shreveport, Shreveport, Louisiana

## Abstract

**Purpose:**

In stereotactic body radiation therapy (SBRT), patient motion during treatment may significantly impact the tumor control probability. In this study, we performed an analysis of patient outcomes to determine the clinical robustness of our lung SBRT protocols against patient motion and to assess the overall efficacy of our unique treatment delivery technique.

**Methods and Materials:**

Seventy-eight patients treated for 85 primary lung tumors or lung metastases using SBRT were included. An abdominal compression belt was used for respiratory motion management. Prescription doses were 50 to 70 Gy, delivered in 5 fractions. Our standard planning target volume expansion was 5.0 mm. Image guided positional corrections were performed halfway through treatment. Intrafraction shifts were recorded for analysis. Kaplan-Meier analysis was performed to determine the 2-and 3-year local control (LC) and overall survival. Adverse effects were evaluated using the National Cancer Institute Common Terminology Criteria for Adverse Events version 6.0.

**Results:**

The median follow-up duration was 25 months (IQR, 13-40 months). Course-averaged intrafraction patient motion ranged from 1.2 to 8.2 mm (median, 3.5 mm; IQR, 2.5-4.0 mm). The 2-and 3-year LC (95% confidence interval) were 97.6% (83.9%-99.7%) and 94.0% (77.3%-98.5%), respectively. The 2-and 3-year overall survival were 77.3% (65.8%-87.8%) and 69.6% (56.8%-79.3%), respectively. The 2 local recurrences were observed at 22 and 31 months post-SBRT. They were associated with the 33rd largest and the smallest average repositioning shifts in the study. No adverse events of grade ≥3 were observed over the study interval.

**Conclusions:**

The excellent LC rates observed irrespective of patient positioning uncertainty were demonstrative of the clinical robustness of our SBRT workflow and may support future studies investigating planning target volume margin reduction. High efficacy and low incidence of adverse events support the implementation of this workflow in clinics developing SBRT programs. Similar study designs may be used to assess alternative SBRT workflows.

## Introduction

Stereotactic body radiation therapy (SBRT) has become a key treatment option for localized primary lung tumors and oligometastases in the thoracic cavity.^1^, ^2^, ^3^, ^4^, ^5^, ^6^, ^7^, ^8^, ^9^, ^10^ The use of steep gradients and high biological effective doses in lung SBRT places an emphasis on treatment delivery accuracy and patient immobilization.^11^, ^12^, ^13^, ^14^ Additionally, thoracic and abdominal tumors may move significantly with respiratory motion.^12^^,^^13^^,^^15^ Lung tumor movement presents clinical challenges that can drastically affect the delivered dose to the patient.^12^^,^^13^^,^^16^, ^17^, ^18^, ^19^, ^20^

To address these challenges, both patient immobilization and respiratory motion management strategies are warranted for lung SBRT.^21^ However, the choice of motion management techniques can significantly affect treatment time and may impact patient outcomes.^12^^,^^13^^,^^22^ Despite the known difficulties in respiratory motion management, local control (LC) rates over 90% have been reported in the literature.^7^^,^^9^^,^^23^^,^^24^

Numerous beam delivery, motion management, and immobilization strategies are in clinical use today, introducing significant heterogeneity across the field. Additionally, as the choice of technique may impact treatment efficacy, heterogeneity and inconsistency in reporting methods may impair portability of reported results to clinical settings. There is, thus, a need for clear reporting and individualized assessment to provide insight into expected results for the specific clinical workflow.

At our facility, we implemented a pneumatic abdominal compression system for respiratory motion management in lung SBRT, along with a treatment delivery scheme using intrafraction repositioning between volumetric dose repaintings. This beam delivery technique was adapted from our proton beam therapy program, where dose repainting is used to mitigate respiratory motion effects.^25^, ^26^, ^27^ The use of volumetric dose repainting also allows for execution of intrafraction positioning shifts to mitigate patient motion during treatment with minimal perturbation to the overall dose distribution. Though intrafraction repositioning is widely used in SBRT,^28^, ^29^, ^30^ repainting techniques are nonstandard in the photon therapy setting.

The purpose of this study was to assess the efficacy of our unique treatment delivery technique in lung SBRT. To assess the robustness of our protocols against intrafraction patient motion, we compiled on-treatment positioning data and follow-up reports to determine if there is a correlation between the magnitude of patient motion during treatment delivery and LC rates. Findings from this study may be portable to clinical practices that adopt similar workflows and can serve as a template for evaluating clinical protocols with continued technological advancements in radiation oncology.

## Methods and Materials

### Patient selection

Patients treated for lung tumors with SBRT between 2018 and 2023 were included in this IRB-exempt retrospective study. All patients were treated using forced shallow breathing via pneumatic abdominal compression for respiratory motion management. A minimum 6-month follow-up interval with accompanying computed tomography (CT) or positron emission tomography (PET)/CT imaging to assess response was required. Treatment of both primary tumors and lung metastases was included. A minimum prescription dose of 50 Gy delivered in 5 fractions to at least 95% of the internal planning target volume (iPTV) was required. Exclusion criteria were treatment of hilar or ultracentral tumors, treatments of recurrent tumors, use of nonstandard treatment techniques due to tolerance or compliance concerns, and postresection SBRT.

### CT simulation and treatment planning

Patients were simulated on a Big Bore RT CT simulator (Philips) in the head-first supine position. The immobilization setup used a Wing Board with a Vac-Lok vacuum bag mounted in the board (CQ Medical). The Body Pro-Lok ONE respiratory belt (BPL1, CQ Medical) was placed on the patient’s upper abdomen and indexed to the table. The belt was inflated to provide abdominal compression, which facilitated forced shallow breathing for respiratory motion management. A knee wedge was placed under the patient for comfort.

Conventional 3-dimensional (3D) and 4-dimensional respiration-correlated CT (4DCT) scans were acquired. Respiratory signals for 4DCT reconstruction were acquired using the C-RAD Sentinel surface monitoring system (C-RAD AB). The conventional scan and the average and maximum intensity projections generated from the 4DCT were exported to the RayStation treatment planning system, versions 7 through 11A (RaySearch Laboratories AB). All dose calculations were performed on the average intensity image set.

Physicians contoured the internal gross tumor (iGTV) and clinical target volumes (iCTV) using the maximum intensity projections. iPTVs were generated using a geometric expansion of the iGTV or iCTV. The clinical standard expansion was a 5.0 mm isotropic margin, though 2 cases used an isotropic 4.0 mm margin and 2 used an isotropic 3.0 mm margin to improve nearby organ sparing (ribs and pericardium). Organs at risk (OARs) were contoured on the average intensity image set.

Treatment plans were generated using a coplanar volumetric modulated arc therapy technique. Six MV or 6 MV flattening filter free beams were used. Following plan approval, the treatment beam monitor units were scaled by 50%, and the beams were duplicated to form 2 symmetric dose halves. Using this technique allowed for volumetric dose repainting, in which half of the dose was delivered, followed by intrafraction imaging and repositioning prior to delivering the second half of the fraction dose, while minimizing dose perturbation from repositioning shifts. Plans used 4 to 6 total coplanar arcs to deliver the prescription dose of 50 to 70 Gy to 95% to 98% of the iPTV. OAR constraints for planning were sourced from Timmerman (2022).^31^

### Treatment delivery

Patients were treated on an Elekta Versa HD (Elekta AB) linear accelerator with 5.0 mm multileaf collimators and a 6-degree-of-freedom couch. Patients were treated with the same indexing positions and compression levels as were used in CT simulation.

**Figure 1:**
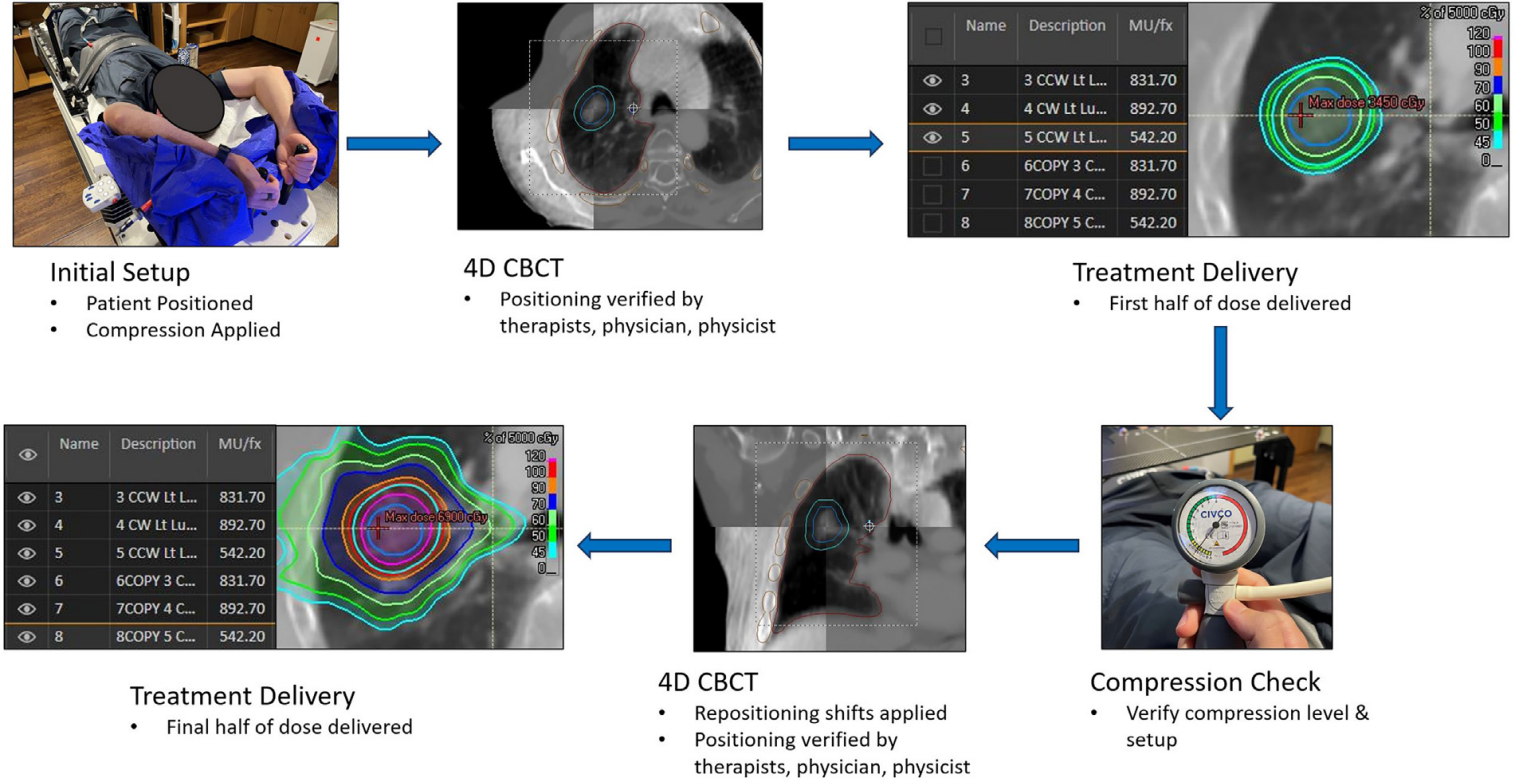
Lung stereotactic body radiation therapy treatment workflow. Four-dimensional cone beam computed tomography (4D CBCT) is used for image guided patient positioning prior to treatment delivery and at the midpoint between the symmetric dose repaintings.

A 4D cone beam CT was used for initial image guided patient setup and for intrafraction repositioning between symmetric dose repaintings (Fig. 1). The magnitude of intrafraction repositioning shifts was recorded for each treated fraction. The repositioning shift magnitude was calculated for each patient as the average total 3D displacement. The time from initiation of patient setup to completion of beam delivery was recorded for analysis.

### Patient outcomes and response assessment

The primary endpoint was local tumor control. LC was defined as freedom from progression within the treated field as determined using CT or PET/CT imaging following RECIST 1.1 criteria and changes in FDG avidity, respectively. Overall survival (OS) was recorded as a secondary endpoint. Patients were scheduled for follow-up assessment and imaging every 3 to 6 months until reaching 5 years post-SBRT, at which point patients were screened on an as-needed basis. Physician-reported adverse effects were assessed using the National Cancer Institute Common Terminology Criteria for Adverse Events version 6.0.

### Data analysis

Descriptive statistics were performed to assess measures of central tendency and dispersion. The 2-and 3-year LC and OS rates were determined using Kaplan-Meier analysis. Subgroups were compared using the log-rank test. Cox proportional hazards modeling was used to perform univariate and multivariate analysis. Statistical significance was taken as *P* <.05. All analyses were performed in STATA version 18.0 (StataCorp LLC).

## Results

Response was assessed for 78 patients with 85 total treated tumors. A summary of patient and tumor characteristics is presented in Table 1. Seventy-two tumors were primary non-small cell lung cancer (NSCLC), while the remaining 13 were metastases from other primaries. The median follow-up interval was 25 months post-SBRT (range, 6-70 months; IQR, 13-40 months). Forty-one tumors (48.2%) were treated using a simultaneous integrated boost to the iGTV or iCTV up to 120% of the primary prescription dose. Forty patients (51.3%) received systemic therapy following SBRT.

**Table 1:** Summary of patient characteristics

Characteristic	Variable	Number of patients or tumors	Percentage of population
Age (y)	Median 73 y IQR, 67-77 y		
Sex assigned at birth (N = 78)	Male	32	41.0
	Female	46	59.0
KPS at treatment (N = 85)	50-60	7	8.2
	70-80	34	40.0
	90-100	44	51.8
NSCLC histology (N = 72)	Adenocarcinoma	38	52.8
	Squamous cell carcinoma	23	31.9
	NOS	11	15.3
NSCLC TNM stage (N = 72)	T1	55	76.4
	T2	11	15.3
	NOS	6	8.3
Primary tumor site (N = 85)	Lung	72	84.7
	Colorectal	6	7.1
	Cervical	3	3.5
	Neuroendocrine	2	2.4
	Renal	1	1.2
	Sarcoma	1	1.2
Tumor location (lobe) (N = 85)	RUL	33	38.8
	LUL	22	25.9
	RML	3	3.5
	RLL	18	21.2
	LLL	9	10.6
Prescription dose (with SIB) (N = 85)	70 Gy/5 Fx	2 (0)	2.4
	60 Gy/5 Fx	41 (14)	48.2
	55 Gy/5 Fx	4 (4)	4.7
	50 Gy/5 Fx	38 (23)	44.7
Systemic therapy (post-SBRT) (N = 85)	No	45	52.9
	Yes	40	47.1

*Abbreviations:* Fx = fractions; KPS = Karnofsky performance status; LLL = left lower lobe; LUL = left upper lobe; NSCLC = non-small cell lung cancer; NOS = not otherwise specified; RLL = right lower lobe; RML = right middle lobe; RUL = right upper lobe; SBRT = stereotactic body radiation therapy; SIB = simultaneous integrated boost.

Course-averaged intrafraction 3D repositioning shifts ranged from 1.2 mm to 8.2 mm (median, 3.5 mm; IQR, 2.5-4.0 mm). Seven patients (8.2%) had average 3D shifts greater than 5.0 mm over their treatment course. The median time from initiation of patient setup to treatment completion was 31 minutes (IQR, 27-35 minutes).

Two patients developed local recurrence as determined with FDG-PET imaging at follow-up. One patient recurred at 22 months post-SBRT, while the other recurred at 31 months. A summary of their individual characteristics is listed in Table 2. The patient recurring at 22 months had the 33rd largest average 3D repositioning shift in the study, whereas the other had the smallest average shift. Both tumors were in the upper lobes (left upper and right upper, respectively).

**Table 2:** Demographics and treatment information for the 2 cases with local recurrence after SBRT

Time to recur (mos)	Average 3D repositioning shift (mm)	KPS	Tumor	Systemic therapy?	Rx dose	PTV margin
22	3.8	100	T1cN0M0 NSCLC (NOS)	None	60 Gy/5 Fx, 95% iPTV (no SIB)	5.0 mm
31	1.2	100	T2aN0M0 NSCLC (adenocarcinoma)	Post-SBRT	60 Gy/5 Fx, 95% iPTV (no SIB)	5.0 mm

*Abbreviations:* 3D = 3-dimensional; Fx = fractions; iPTV = internal planning target volume; KPS = Karnofsky performance status; NOS = not otherwise specified; NSCLC = non-small cell lung cancer; PTV = planning target volume; SBRT = stereotactic body radiation therapy; SIB = simultaneous integrated boost.

**Figure 2:**
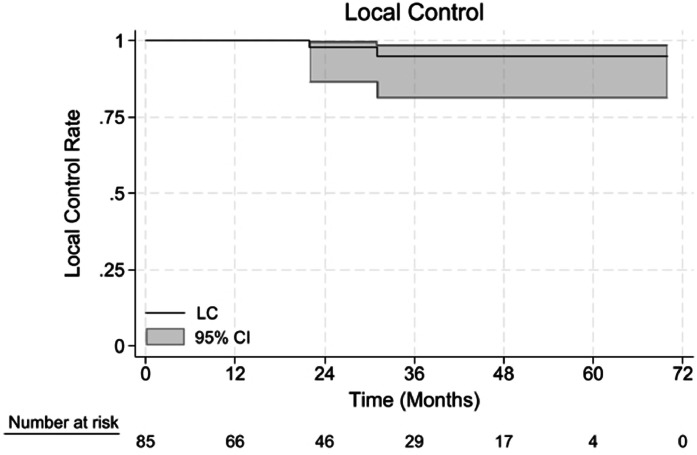
Local control (LC) over interval following stereotactic body radiation therapy treatment. *Abbreviation:* CI = confidence interval.

There was no observed relationship between the average 3D repositioning shifts and LC rates. The overall 2- and 3-year LC (95% confidence interval [CI]) were 97.6% (83.9%-99.7%) and 94.0% (77.3%-98.5%), respectively (Fig. 2).

**Figure 3:**
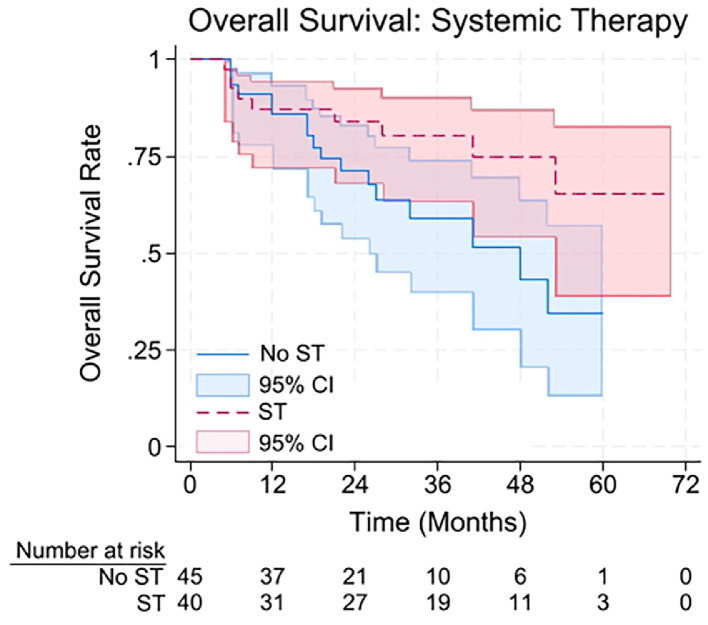
Overall survival for patients treated with or without systemic therapy (ST) following stereotactic body radiation therapy. *Abbreviation:* CI = confidence interval.

The 2-and 3-year OS rates (95% CI) were 77.3% (65.8%-87.8%) and 69.6% (56.8%-79.3%), respectively. There was a marginal, but not statistically significant, dependence of OS on the use of systemic therapy following SBRT (Fig. 3, *P* = .057). The 2-and 3-year OS without systemic therapy were 70.9% (95% CI, 53.2%-82.9%) and 58.0% (95% CI, 38.2%-73.5%), respectively. With systemic therapy, patients had respective 83.9% (95% CI, 67.5%-92.5%) and 80.4% (95% CI, 62.9%-90.2%) OS at 2 and 3 years.

**Figure 4:**
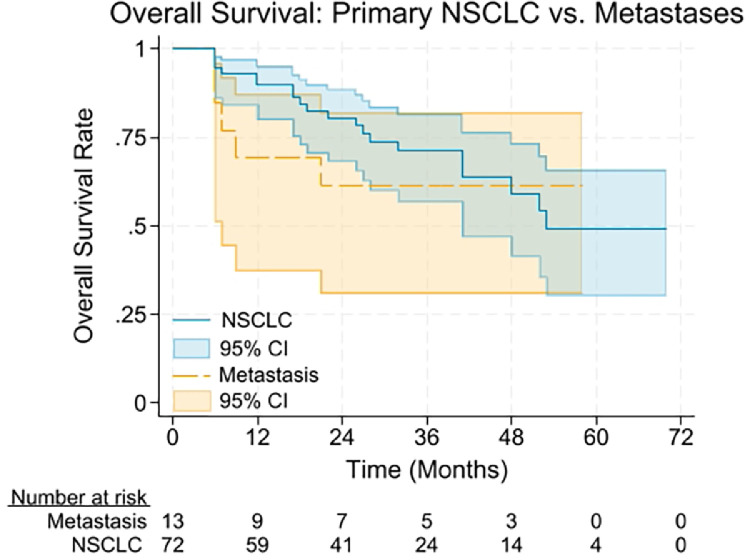
Overall survival for patients treated using stereotactic body radiation therapy for primary non-small cell lung cancer (NSCLC) or lung metastases. *Abbreviation:* CI = confidence interval.

Multivariate analysis revealed that tumor location (*P* = .619), treatment of a primary tumor versus lung metastasis (Fig. 4, *P* = .535), Karnofsky performance status (*P* = .567), patient age (*P* = .398), target volume (*P* = .275), and prescription dose (*P* = .127) did not significantly affect OS. The tumor histology (adenocarcinoma versus squamous cell carcinoma) did not affect OS for patients with biopsy-proven NSCLC (Fig. E1).

Over the study interval, no grade ≥3 adverse events (AEs) were reported for the cohort. Six grade 2 AEs were reported: esophagitis (1), dyspnea (1), pleural effusion (1), pneumonitis (1), cough (1), and rib fracture within the treated field (1). The most common acute grade 1 AEs included fatigue (11), dyspnea on exertion (8), and cough (8). Complete AE data are summarized in the supplementary material (Table E1).

## Discussion

SBRT remains one of the most logistically and mechanically demanding radiation therapy techniques available today.^11^^,^^32^ Accurate patient positioning and motion management are requisites for safe SBRT delivery.^14^^,^^21^ With continued technological advancements, significant planning target volume (PTV) margin reduction may be feasible for current SBRT delivery techniques.^33^, ^34^, ^35^, ^36^, ^37^ However, the heterogeneity in treatment techniques in use today limits the portability of reported results to clinical settings. In this study, we reported clinical results for our unique treatment delivery scheme and used intrafraction patient motion and follow-up data to evaluate the robustness of our current protocols against positioning uncertainty. To our knowledge, this is the first study to directly assess the impact of intrafraction patient motion on tumor control rates in lung SBRT.

Despite several patients having intrafraction motion greater than 5.0 mm, the only 2 local recurrences in this study were in patients with relatively small positioning shifts, indicating that LC was not linked to the detected intrafraction patient motion for our study cohort. Our results, thus, demonstrated that LC was achieved even under large positional uncertainties, demonstrating the clinical robustness of our protocols. Additionally, no grade >2 AEs were reported over the study interval, indicating an acceptable balance between tumor control and safety profiles.

Considering the breadth of literature reporting 80% to 95% 2-year LC rates,^1^, ^2^, ^3^^,^^5^^,^^6^^,^^24^ our 97.6% and 94.0% 2-year and 3-year control rates demonstrate excellent clinical outcomes with our workflow. However, our consistent use of enhanced localization techniques such as on-treatment 4D cone beam CT and intrafraction repositioning may reduce the probability of a geometric miss as compared to historical techniques,^33^^,^^34^^,^^38^ and may better represent the current standard of care.

It has been shown that current treatment techniques can decrease the necessary PTV margin below the common 5.0 mm standard.^33^^,^^34^ Previous work used intrafraction shifts to calculate PTV margin requirements in lung SBRT for our clinical workflow.^34^ The findings revealed that 4.5 mm inferior-superior and 4.0 mm lateral and anterior-posterior margins were suitable for lung SBRT treatment with abdominal compression and intrafraction repositioning between volumetric dose repaintings.^34^ Therefore, the high LC rates could be a consequence of using planning margins that may be larger than necessary for our procedures, as indicated by durable tumor control being achieved for patients with large repositioning shifts.

Given the excellent control rates for patients irrespective of the magnitude of positional uncertainty and the low incidence of AEs reported in lung SBRT,^2^^,^^3^^,^^5^^,^^6^^,^^39^ including in this study, there is merit to retaining standard 5.0 mm planning margins, as reducing margins may carry additional risk of underdose with relatively little benefit to the patient. However, in situations where margin reduction may be needed to spare nearby OARs, such as abutting ribs, heart, or large bronchi, the results indicate that acceptable tumor control may still be achieved. The findings of this study may support future research investigating tumor control rates with varied PTV margins in a controlled trial setting.

The OS measured in this study was representative of the survival rates reported in the literature.^1^^,^^5^^,^^6^^,^^23^^,^^39^^,^^40^ Results showed that systemic therapy had the biggest impact on OS. Though this suggests that systemic therapy can improve survival when used in conjunction with SBRT, its effect was not statistically significant for our cohort. However, the results were not stratified by type of systemic therapy or specific regimen due to limited statistical power, which may mask some differences between therapeutic agents. Further intercomparisons between different systemic therapies may be merited for a larger data set to fully assess the impact on OS.

OS was also not significantly affected by whether patients were being treated for primary NSCLC or lung metastases; however, the number of metastases treated in this study was small as compared to treatments for NSCLC. Other studies have reported mixed results when comparing OS for patients treated with SBRT for primary NSCLC and lung metastases.^41^ Further investigations with larger cohorts are warranted to better characterize the effects.

Our use of nonstandard techniques such as volumetric dose repainting and abdominal compression limits the generalizability of the results to our specific workflow. However, the results demonstrate that using this workflow yields excellent clinical outcomes. Furthermore, the 31-minute median treatment time demonstrates that we maintain high workflow efficiency, despite our use of volumetric dose repainting. Our previous work showed that volumetric dose repainting reduced 5-fraction lung SBRT PTV margin requirements by 0.73 ± 0.07 mm,^34^ using the calculation method proposed by Janssen et al.^42^ Additionally, intrafraction repositioning may effectively reduce the impact of any patient settling effects during treatment, while providing potential dosimetric benefits, such as reductions in the interplay effect.^16^^,^^17^^,^^19^^,^^43^ For modulated treatment plans, the use of symmetric dose repaintings allows for intrafraction positioning corrections to be performed with minimal impact to the overall dose distribution.

Excellent patient outcomes demonstrate the clinical viability of this treatment workflow. Although unique, clinics utilizing or adopting similar techniques may expect results comparable to those presented in this work. However, the results presented here may not be directly generalizable to alternative treatment protocols, though similar analyses may be performed to assess their robustness and efficacy. This study may serve as a template for further evaluation of other SBRT protocols.

The limitations of outcomes analysis in lung SBRT have been thoroughly covered in the literature.^6^^,^^24^^,^^40^^,^^44^, ^45^, ^46^ There are other limitations specific to this study that should be considered:

1. Patients presented with a range of primary tumor types and performance status at treatment (Table 1), introducing heterogeneity into the cohort.
2. Nearly half of the patients were treated with systemic therapy following SBRT, which may have masked the impacts of intrafraction shifts on tumor control, leading to potential overestimation of tumor control rates. Treatment with systemic therapy also impacted assessment of treatment safety, as effects from SBRT may not be reliably decoupled from those of systemic therapy.
3. The low number of local failures in this cohort limits reliable stratification of results and identification of underlying factors predictive of local recurrence.
4. Our data were dominated by treatments for stage T1 or T2 NSCLC. Therefore, the results are most applicable to the treatment of early-stage NSCLC, and interpretation in the context of lung metastases is limited for this cohort.

Future studies may benefit from further outcome analysis for the treatment of lung metastases. Nevertheless, the results demonstrate excellent patient outcomes across a variety of clinical scenarios for which lung SBRT may be indicated.

## Conclusions

This study demonstrates that our unique workflow achieves excellent tumor control outcomes in lung SBRT irrespective of the magnitude of patient positioning uncertainty. The combination of volumetric dose repainting and forced shallow breathing via abdominal compression is clinically robust against intrafraction patient motion. Given successful tumor control despite average positioning uncertainties up to 8.2 mm, planning margin reduction may be feasible for our workflow. However, our excellent clinical outcomes with a low incidence of grade 2 AEs also provide merit to maintaining our standard protocols. Other facilities implementing similar workflows may expect comparable results to those presented here. This work may serve as a template for other facilities evaluating the robustness of their clinical workflows.

## Disclosures

Yohan A. Walter and Daniel B. Speir report a relationship with Medtec LLC (CQ Medical) that includes: consulting or advisory, funding grants, and travel reimbursement. Hsinshun T. Wu and Chiachien J. Wang report a relationship with Medtec LLC (CQ Medical) that includes: funding grants. William E. Burrell and Daniel B. Speir report a relationship with C-RAD AB that includes: consulting or advisory. Joseph P. Dugas reports a relationship with the American Association of Physicists in Medicine that includes: board membership. Mitchell Wolden reports a relationship with the American Physical Therapy Association Academy of Education and the American Physical Therapy Association of North Dakota that includes: funding grants. The other authors declare that they have no known competing financial interests or personal relationships that could have appeared to influence the work reported in this paper.

## Appendix Supplementary materials

Supplementary Materials Image, application 1

## Footnotes

Supplementary material associated with this article can be found in the online version at doi:10.1016/j.adro.2026.102064.
