# Supplementary material for: Robustness of Tumor Control Against Intrafraction Patient Motion in Lung Stereotactic Body Radiation Therapy
Source: Adv Radiat Oncol. 2026 Apr 17;11(7):102064. doi: 10.1016/j.adro.2026.102064 (PMC13253121; doi:10.1016/j.adro.2026.102064)
Supplement: Supplementary Materials [file mmc1.docx]

# **Supplementary Material: “**Robustness of Tumor Control Against Intrafraction Patient Motion in Lung Stereotactic Body Radiation Therapy”


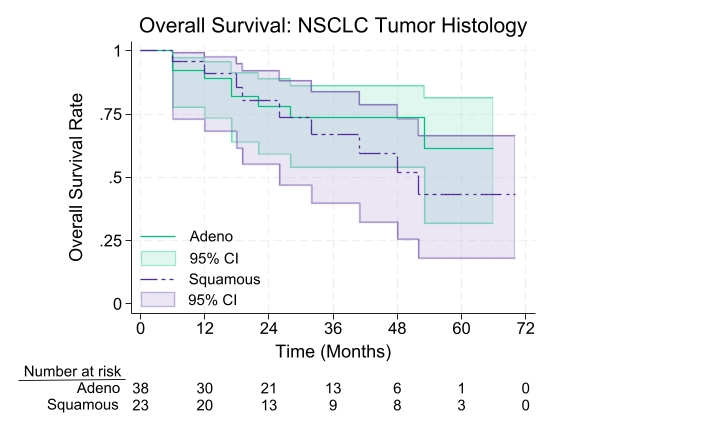


Figure E.1: Overall survival for patients treated with SBRT for primary NSCLC, separated by biopsy-proven tumor histology.

Table E.1: Physician-reported adverse events reported over the study interval based on National Cancer Institute Common Terminology Criteria for Adverse Events (CTCAE) version 6.0.

| **Body site** | **Adverse effect** | **Grade (CTCAE v6.0)** | | | |
| --- | --- | --- | --- | --- | --- |
|  |  | **1** | **2** | **3** | **≥4** |
| Gastrointestinal | *Esophagitis* | 0 | 1 | 0 | 0 |
| Respiratory | *Cough* | 8 | 1 | 0 | 0 |
|  | *Dyspnea* | 8 | 1 | 0 | 0 |
|  | *Hemoptysis* | 1 | 0 | 0 | 0 |
|  | *Pleural Effusion* | 1 | 1 | 0 | 0 |
|  | *Pneumonitis* | 0 | 1 | 0 | 0 |
| Skin | *Erythema* | 1 | 0 | 0 | 0 |
| Miscellaneous | *Bone Fracture* | 0 | 1 | 0 | 0 |
|  | *Fatigue* | 11 | 0 | 0 | 0 |
|  | *Pain* | 1 | 0 | 0 | 0 |
| **Total** |  | ***31*** | ***6*** | ***0*** | ***0*** |
